# Experiences with and perspectives on firearm injury prevention among emergency medical services clinicians

**DOI:** 10.1186/s12873-025-01241-9

**Published:** 2025-06-02

**Authors:** Amanda J. Aubel, Avery Baldwin, Amy Barnhorst, Angela M. Bayer

**Affiliations:** 1 Violence Prevention Research Program, Department of Emergency Medicine, University of California Davis School of Medicine, 2315 Stockton Blvd, Sacramento, CA 95817 USA; 2 California Firearm Violence Research Center at UC Davis, 2315 Stockton Blvd, Sacramento, CA 95817 USA; 3 Reno Fire Department, City of Reno, 1 E 1st St, Reno, NV 89501 USA; 4 Department of Psychiatry and Behavioral Sciences, University of California Davis School of Medicine, 2315 Stockton Blvd, Sacramento, CA 95817 USA

**Keywords:** Gun violence, Emergency medical services, Risk assessment, Firearm, Injury prevention, Suicide

## Abstract

**Background:**

Firearm-related injury is a significant public health problem in the United States. Emergency medical services (EMS) personnel are uniquely positioned to recognize and counsel individuals at risk, but little is known about their firearm screening and counseling practices, experience with firearms, and training needs. To address these knowledge gaps and inform training efforts, this study examined the current and potential role of EMS clinicians in firearm injury prevention.

**Methods:**

A 22-item survey was e-mailed to EMS agencies, predominantly in California and Nevada. EMS clinicians who had worked 5 + shifts in the previous three months were eligible to participate. Question topics included: participant characteristics, recent encounters with at-risk patients, career exposure to firearm risk situations, experience with firearms, and training on firearm injury prevention. Descriptive statistics were calculated, and write-in responses were analyzed thematically.

**Results:**

Among 234 participants, 75% reported that at least some of their calls in the past three months involved someone at risk of firearm-related injury. Among those who responded to at-risk patients, 47% reported that they never asked these patients about firearm access and 88% said that they did not provide them with education/counseling on firearm injury prevention. 76% were at least somewhat worried about being injured by a firearm while on duty, and 19% reported having had a firearm drawn on or used against them during a call. Participants reported being on duty without law enforcement when firearms were accessible to suicidal patients (70%), to children (47%), and in domestic violence situations (49%). Approximately 70% reported current or previous firearm ownership, and 82% felt comfortable handling firearms. More than one-third had not received medical education on firearm injury prevention, and 85% expressed interest in future training.

**Conclusions:**

As the sole health care providers for many patients and given their unique prehospital perspective, with additional training, EMS clinicians can expand their role in firearm injury prevention. Training should: respect the right to own firearms; prioritize a risk-based approach to firearms screening; highlight interventions for reducing firearm injury risk; address the increased risk of firearm injury among EMS clinicians; and align with local policies, including those involving law enforcement.

**Clinical trial number:**

Not applicable.

**Supplementary Information:**

The online version contains supplementary material available at 10.1186/s12873-025-01241-9.

## Background

Firearm-related injuries and deaths are a significant public health burden in the United States (US). In 2022, there were 48,204 firearm-related deaths nationwide (14.5 per 100,000 people), of which 56% were suicides and 41% were homicides [1]. This exceeded the death rates for falls (14.4 per 100,000) and motor vehicle crashes (13.4 per 100,000) in the same year [1]. Despite evidence that firearm access substantially increases the risk of suicide, homicide, and unintentional firearm injury, [2 — 5] a growing number of US adults report owning and carrying firearms. About 2 in 5 US households have a firearm, and an estimated 7.5 million people became new firearm owners between January 2019 and April 2021 [6]. Research has also estimated that in 2019, approximately 6 million adults carried a loaded handgun daily, twice as many as in 2015 7.

There is growing recognition and support for the role of physicians and other hospital-based clinicians in preventing, not just treating, firearm injuries. Similar to other health and safety issues, health care practitioners are uniquely positioned to identify patients at risk for firearm-related injury and provide counseling or other interventions to reduce the risk of harm [8]. Across specialties, health professional societies and majorities of physicians believe that firearm safety counseling falls within their scope of practice [9 — 16]. Much of the public is also supportive of these discussions. Approximately two-thirds of US adults, including 54% of firearm owners, think that it is at least sometimes appropriate for health professionals to talk with their patients about firearm safety [17]. Levels of public support for such conversations are higher when patients or their family members have risk factors for harm, such as suicidal thoughts, substance misuse, or children in the home [18, 19].

Efforts to train health care practitioners in firearm injury prevention have, to date, predominantly focused on physicians and other hospital-based personnel; [20— 22] however, prehospital clinicians may also be able to make important contributions towards preventing firearm injuries among patients. Specifically, emergency medical services (EMS) clinicians have unique access to patients in their homes and communities, presenting a significant opportunity to assess risk and provide individualized and tailored recommendations to reduce risk. Unlike hospital-based clinicians, EMS clinicians are often able to directly observe many aspects of a patient’s life, including their living conditions, social interactions, and potentially, access to firearms. Furthermore, for patients not ultimately transported to the hospital, an EMS response may be the highest level of medical care they receive. EMS may be the sole responder on calls when resources such as law enforcement are not available, and some of these patients may be suicidal, agitated, or in a crisis. Understanding the risks imposed by firearms and how to mitigate those risks is therefore important not only for keeping their patients safe, but also for ensuring the safety of EMS personnel and others responding to the scene.

Previous research has examined EMS providers’ response to incidents of firearm violence, particularly active shootings, [23 — 25] and their exposure to weapons and violence in the course of their duties [26 — 31]. However, with few exceptions, [32, 33] there has been little research exploring the role EMS clinicians can play further “upstream” by helping prevent firearm injuries before they occur. One study of 229 US EMS clinicians laid important groundwork by examining the feasibility of EMS clinicians delivering lethal means safety counseling (LMSC) and initiating extreme risk protection orders (ERPOs) – two key strategies for preventing firearm-related injury [32, 33]. Reported use (14.8% had delivered LMSC; 4.4% had initiated an ERPO) slightly exceeded past training (7.4% had received LMSC training; 2.2% had received ERPO training), suggesting a need for these tools in the field. In addition, interest in further training was high, with 67.7% expressing interest in learning how to counsel individuals on secure firearm storage.

Beyond these preliminary insights, much remains unknown about EMS clinicians’ encounters with patients at risk for firearm-related injury, as well as their counseling practices and training needs. To fill this gap, the current study explores EMS clinicians’ experiences and perspectives related to firearms, firearm-related injuries, and firearm injury prevention. Findings can inform the development of future training for EMS clinicians, in turn broadening the cadre of health care professionals who are equipped and ready to reduce the risk of firearm injuries among patients and their families.

## Methods

This study was conducted as part of The BulletPoints Project, a program funded by the State of California and led by the California Firearm Violence Research Center at the University of California, Davis (UC Davis). The BulletPoints Project teaches medical and mental health care practitioners strategies to reduce the risk of firearm-related injuries in their patients [20].

### Study design and data collection

We conducted an online survey of EMS clinicians between February and April 2022. EMS clinicians who had worked or volunteered during five or more shifts in the previous three months were eligible to participate. We used several sampling techniques to recruit participants. First, we compiled a list of fire-based and non-fire-based EMS departments and state EMS organizations in California and Nevada (due in part to our funding and existing relationships with EMS departments in these states) and national EMS groups. We used publicly accessible, online directories and Google searches to identify EMS agencies and their contact information. We sent an e-mail with the survey link to each agency’s Director, Medical Director, EMS Program Manager or Coordinator, or other contact person, explaining the purpose of the study and asking them to share the survey with the EMS personnel in their department. Initial e-mails were sent to 306 fire-based EMS departments, 38 non-fire-based EMS departments, and 4 state and 4 national EMS groups; follow-up e-mails were sent approximately 5–6 weeks later. Then, to increase our sample size, we invited our colleagues at the UC Davis Violence Prevention Research Program to share the survey with their EMS contacts, regardless of where they lived or worked.

All participants read informed consent language, and survey initiation constituted their consent. Surveys were anonymous and did not collect any personally identifiable information. The study was deemed exempt by the UC Davis Institutional Review Board.

### Survey instrument

The survey was conducted using Qualtrics and included 22 questions. Eligibility was assessed with an initial screening question: “In the last 3 months, did you work 5 or more shifts as an EMS provider?” Those who answered affirmatively were subsequently asked about their: encounters with patients at risk for firearm-related injury and their screening and counseling practices in the last three months; career exposure to firearm risk situations while on duty and lifetime experience with firearms; previous training related to firearms and firearm injury prevention; and interest in future training. We also collected information on participants’ age, gender, current state of residence, state in which they grew up, type of EMS agency for which they primarily work/volunteer, and highest level of medical certification. The full survey instrument can be found in the Appendix.

### Data analysis

We calculated standard descriptive statistics (counts and percentages) for the quantitative data. For the two open-ended questions (“What other firearm injury prevention topics would you be interested in learning about?” and “Is there anything else you’d like to share with us?”), we reviewed participants’ qualitative responses and identified distinct and important categories or themes, using an inductive approach. All analyses were conducted in Microsoft Excel or Stata, version 18.0 (StataCorp).

## Results

### Participant characteristics

A total of 409 people answered the screening question, of whom 352 (86.1%) were eligible for the study. Of those eligible, 118 (33.5%) did not complete the survey and were thus excluded from analyses; this included 26 individuals (7.4%) who did not answer any questions after screening, 65 (18.5%) with more than 50% missing data, and 27 (7.7%) with 1–50% missing data. The final sample size was 234. Most participants were 25 to 34 years old (34.6%) or 35 to 44 years old (26.5%), and 70.9% were male (Table 1). 71% of participants reported paramedics as their highest level of medical training. 46% of participants were fire department-based, followed by 45.3% who worked for a private EMS service. 73% of participants currently resided in California and 20.9% lived in Nevada.

**Table 1:** Sociodemographic characteristics of survey participants (*n* = 234)

	Count	Percent
Age, in years		
≤ 24	29	12.4%
25–34	81	34.6%
35–44	62	26.5%
45–54	45	19.2%
≥ 55	17	7.3%
Gender		
Male	166	70.9%
Female	61	26.1%
Other	1	0.4%
Preferred not to answer	6	2.6%
Highest level of medical training		
First Responder	1	0.4%
Emergency Medical Technician (EMT)	35	15.0%
Advanced Emergency Medical Technician (AEMT)	23	9.8%
Paramedic	166	70.9%
Registered Nurse (RN)	8	3.4%
Physician Assistant (PA)	1	0.4%
Primary work service		
Fire department-based EMS service	107	45.7%
Private EMS service	106	45.3%
Municipal, county, or government-based EMS service	11	4.7%
Hospital-based EMS service	7	3.0%
Other	3	1.3%
State of residence		
California	170	72.7%
Nevada	49	20.9%
Other	15	6.4%
State of high school graduation		
California	174	74.4%
Nevada	31	13.2%
Other	29	12.4%

### Recent encounters with at-risk patients and conversations about firearms

In general, nearly half of participants reported being most concerned about firearm-related homicide and interpersonal violence among their patients (48.7%), followed by suicide or self-harm (38.0%) and unintentional injuries (13.3%) (Table 2). When asked about encounters during the previous three months, 74.8% of participants reported that at least some of their calls involved a patient or other person at risk of causing or sustaining a firearm-related injury; for most participants (66.7%), this occurred in less than one-quarter of their calls. Among those who responded to at-risk patients in the past three months, nearly half (46.9%) reported never asking these patients about their access to firearms or the presence of firearms, and 88.0% had not provided them with education or counseling on firearm injury prevention. The most common reasons for not asking at-risk patients about their access to firearms were because participants did not think that firearm access was relevant to a patient’s risk of injury or death (32.1%), did not think asking about firearm access falls within their professional responsibilities (28.6%), and thought there was nothing they could do for an at-risk patient with a gun (18.8%). However, 59.8% said they felt comfortable or very comfortable counseling their patients about firearm injury prevention.

**Table 2:** Participants’ recent encounters with at-risk patients and conversations about firearms and firearm injury prevention with patients (*n* = 234)

	Count	Percent
Most concerning type of firearm-related harm among patients served		
Homicide or interpersonal violence	114	48.7%
Suicide or self-harm	89	38.0%
Unintentional injury	31	13.3%
Calls involving patients at risk of firearm-related injury, during last 3 months		
None	59	25.2%
A small amount (1–25%)	156	66.7%
A moderate amount (26–50%)	15	6.4%
Many of them (51–75%) or most of them (76–100%)	4	1.7%
Asked at-risk patients about access to firearms, during last 3 months^†^		
None	82	46.9%
A small amount (1–25%)	74	42.3%
A moderate amount (26–50%)	10	5.7%
Many of them (51–75%) or most of them (76–100%)	9	5.1%
Provided firearm injury prevention education or counseling to at-risk patients, during last 3 months^†^		
None	154	88.0%
A small amount (1–25%)	17	9.7%
A moderate amount (26–50%)	2	1.1%
Many of them (51–75%) or most of them (76–100%)	2	1.1%
Comfort level in counseling patients about firearm injury prevention		
Very comfortable	56	23.9%
Comfortable	84	35.9%
Uncomfortable	69	29.5%
Very uncomfortable	25	10.7%
Reasons for not asking at-risk patients about access to firearms*		
I don’t think firearm access is relevant to their risk of injury or death	75	32.1%
I don’t think asking about firearm access is within my professional responsibilities	67	28.6%
There’s nothing I could do for an at-risk patient who has a gun	44	18.8%
I wouldn’t know what to do if the patient had a gun	35	15.0%
I’m worried that the patient will be offended if I ask	33	14.1%
I don’t have time to ask	31	13.3%
I don’t know how to ask about firearm access	25	10.7%
I’m not sure if it’s legal for me to ask	13	5.6%

^†^Among participants who reported seeing at least “a small amount” of patients at risk in the last 3 months (*n* = 175).

*Participants could select more than one response. Thirty-three participants (14.1%) did not select any of the responses

### Career exposure to firearm risk situations and lifetime experience with firearms

Participants reported encountering a variety of firearm risk situations while on duty and in the absence of law enforcement (Table 3). More than two-thirds said they had been physically assaulted by a patient (75.6%), had been on a call in which an at-risk patient had access to a firearm (72.2%), and had cared for a patient with active suicidal ideation or following a suicide attempt who had firearm access (69.7%). About half of participants said they had located a firearm in the possession of an altered or unresponsive patient (57.7%), had been present during a domestic violence situation in which a firearm was present (49.2%), had been on a call where firearms were accessible to children (47.0%), and had handled a firearm during a call (44.4%). Approximately 20% reported having had a firearm drawn on or used against them while on duty. When asked how worried they are, in general, about being injured by a firearm while on duty – whether on purpose or unintentional – 75.6% of participants reported being at least somewhat worried.

**Table 3:** Participants’ exposure to firearms while on duty and over lifetime (*n* = 234)

	Count	Percent	
Situations encountered while on duty when law enforcement was not present, during career*			
Have been physically assaulted by a patient	177	75.6%	
Cared for at-risk patient who had access to firearms	169	72.2%	
Cared for a patient with active suicidal ideation or following a suicide attempt who had firearm access	163	69.7%	
Located a firearm on an altered or unresponsive patient	135	57.7%	
Domestic violence situation with a firearm present	115	49.2%	
Situation when firearms were accessible to children	110	47.0%	
Have handled a firearm	104	44.4%	
Have had a firearm drawn or used against them	45	19.2%	
Level of worry about being injured by a firearm while on duty			
Not worried at all	57	24.4%	
Somewhat worried	134	57.3%	
Worried	33	14.1%	
Very worried	10	4.3%	
Lifetime experience with firearms*			
Own or have owned a firearm	163	69.7%	
Live with someone who owns a firearm, but do not personally own	22	9.4%	
Grew up around firearms	121	51.7%	
Have used a firearm	188	80.3%	
Have used a firearm as part of military service	18	7.7%	
Have never used a firearm	9	3.9%	
Prefer not to answer	12	5.1%	
Comfort level in handling a firearm			
Very comfortable	132	56.4%	
Comfortable	61	26.1%	
Uncomfortable	29	12.4%	
Very uncomfortable	12	5.1%	

*Participants could select more than one response.

Most participants reported prior experience with or exposure to firearms (Table 3). Approximately 70% reported current or previous firearm ownership, 51.7% reported growing up around firearms, and 80.3% reported having previously used a firearm. More than 80% reported feeling comfortable or very comfortable handling firearms.

### Past and future training related to firearms and firearm injury prevention

Nearly three-quarters of participants (73.1%) reported having previously taken a course on the safe handling and use of firearms (Table 4). Half of participants (50.0%) said that firearm injury prevention was addressed in their EMT, AEMT, or paramedic classes, and nearly one-quarter (24.4%) reported receiving continuing education on the topic. However, more than one-third (38.5%) said they had not received any medical or professional education on firearm injury prevention.

**Table 4:** Participants’ past training related to firearms and firearm injury prevention and interest in future training (*n* = 234)

	Count	Percent
Has taken a firearms safety course		
Yes	171	73.1%
No	63	26.9%
Type of medical education received that addressed firearm injury prevention*		
EMT, AEMT, or paramedic classes	117	50.0%
RN, PA, or physician training	2	0.9%
Continuing education courses	57	24.4%
Other professional education	10	4.3%
None	90	38.5%
Interest in future firearm injury prevention training		
Very interested	47	20.1%
Interested	89	38.0%
Slightly interested	62	26.5%
Not interested at all	36	15.4%
Firearm injury prevention topics interested in learning more about*		
What to do if at-risk patients are in possession of firearms	157	67.1%
Evidence-based interventions to prevent firearm injury and death in patients	147	62.8%
How to properly handle a firearm and render it safe	107	45.7%
How to talk with patients about their access to firearms	102	43.6%
How to identify patients at risk of firearm-related injury	97	41.5%
How to counsel patients on safe firearm storage	78	33.3%
Why patients would be prohibited from owning or purchasing firearms	67	28.6%
Other	33	14.1%

*Participants could select more than one response.

EMT = Emergency Medical Technician; AEMT = Advanced Emergency Medical Technician; RN = Registered Nurse; PA = Physician Assistant

The majority of participants (84.6%) reported being at least slightly interested in future training to increase their knowledge and skills in firearm injury prevention counseling (Table 4). With regard to specific topics of interest, participants were most interested in learning more about what to do if at-risk patients have access to firearms (67.1%) and evidence-based interventions to prevent firearm injury and death in patients (62.8%). Other topics of interest included how to properly handle a firearm and render it safe (45.7%), how to talk with at-risk patients about their access to firearms (43.6%), and how to identify patients at risk for firearm-related injury (41.5%). In their write-in responses, participants expressed interest in learning more about de-escalation techniques and legal considerations, including the “legal obligations of EMS in regard to firearms,” “legal boundaries as EMS providers and firearm questioning,” and “the legalities surrounding securing firearms found on scene when transporting a person with a CCW [carry concealed weapon permit] and or law enforcement.” Several participants took this opportunity to request personal protective equipment (i.e., body armor) for EMS personnel.

### Qualitative responses

Participants provided in-depth responses to an open-ended final question that asked whether there was anything else they would like to share. Three key themes emerged from their remarks: (1) need for education; (2) co-response with law enforcement; and (3) support for gun ownership (Table 5). With regard to the need for education, participants acknowledged that firearms can be a “taboo” subject often avoided by health care agencies, which do not discuss it with employees or offer related training. Providing additional education to EMS personnel and, by extension, to the public would bolster awareness, knowledge, and skills and help prevent injuries. As for co-response with law enforcement, participants suggested that the protocol in most areas is for law enforcement to respond first to any situations involving firearms and ensure scene safety prior to EMS entering. As a result, EMS was seen as having a “limited role” in firearm injury prevention, which was conceptualized chiefly as a set of strategies for averting harm when firearms were already determined to be present. Finally, a few participants expressed support for gun ownership, including support for the right to own firearms and for EMS clinicians to carry a concealed weapon.

**Table 5:** Themes and example quotes from participants’ qualitative responses

Theme	Example quotes
Need for education	EMS, hospitals, and law enforcement do a poor job in educating the public on firearm safety. The subject of firearms is so taboo that agencies refuse to talk about, or educate about firearms in the fear that it will be seen as promoting firearms. If more people had training on firearms, less accidents would happen, period. Hiding guns knowledge from people only makes firearms more dangerous.
	Honestly, it has never even occurred to me to ask about access to firearms and now, it seems so obvious that I’m kicking myself. I’ve been a medic for 11 years and have never had a lecture, CE course or anything about firearm injury prevention. That’s staggering.
Co-response with law enforcement	If there is a firearm involved, we would have police determine the area safe prior to our entry to the scene.
	This is what law enforcement is for. Anything like the situations you describe would have them there.
	I hope you understand the limited role most EMS and fire has with firearm prevention. We respond after something happens. PD [police department] is more responsible for people who call because they have concerns. If there’s a gun possibility, EMS and fire will stage (wait until PD deems the scene safe) before entering.
	PD has to be on scene when firearms are involved, and we are called in after the scene is secure.
Support for gun ownership	I feel people have the right to own firearms with the proper education and safety.
	More lives are saved by people trained to use firearms than lost to them.
	Law abiding citizens should have a right to bear arms.
	We should be allowed concealed carry.

## Discussion

This is one of a limited number of studies to examine EMS clinicians’ experiences and perspectives related to firearm injury prevention. Findings suggest that EMS personnel encounter a variety of situations in which firearms pose a risk to both their patients and themselves, but they rarely ask about firearm access or provide education or counseling on firearm injury prevention to at-risk patients. In fact, in their recent calls involving patients whom they perceived to be at risk for firearm-related injury, only 5% of participants reported asking “many” or “most” of these patients about the presence of firearms and only 1% provided “many” or “most” of them with education or counseling. Universal screening for firearms may be impractical and, in many cases, irrelevant to a patient’s health and safety; however, when a patient is at elevated risk, asking about access to firearms can be an important step towards reducing risk of harm.

Interestingly, the most common reasons for not asking at-risk patients about their access to firearms reflect EMS clinicians’ doubts that such discussions are relevant to the patient’s risk of injury or death, within their professional scope, and actionable (i.e., “There’s nothing I could do”). Participants were less often worried about offending patients or not having enough time, barriers frequently reported by physicians and other hospital-based practitioners [11, 34 — 36]. Integrating firearm injury prevention into related training topics and formats already familiar to EMS clinicians may help normalize such conversations and underscore their importance [37]. For example, EMS clinicians frequently encounter intimate partner violence (IPV) and may be the first or only health care providers patients see, particularly when patients refuse transport to the hospital [38, 39]. In response, a clinical guideline was developed to help paramedics recognize IPV and refer patients to appropriate services [40]. Similarly, the role of first responders in identifying and supporting older adults at increased risk of harming themselves or others – often linked to cognitive impairment – has been acknowledged, especially in rural areas [41]. These examples not only illustrate that EMS clinicians can feasibly screen patients for risk factors and provide recommendations or referrals, but they also describe scenarios in which firearm access increases the risk of injury and death (e.g., IPV, cognitive impairment) [42, 43]. Using case studies to demonstrate a risk-based approach to firearms screening and intervention – asking about firearm access only when a patient has risk factors for injury – may help EMS clinicians view firearm injury prevention as a relevant and achievable part of their role. Indeed, research has found that EMS clinicians are more supportive of providing lethal means safety counseling to patients with specific risk factors, such as suicidality, than to patients in general [32].

When designing clinical education and training curricula on firearm injury prevention, it is important to recognize that EMS clinicians differ from hospital-based clinicians in at least two notable ways. First, EMS clinicians may be at particularly high risk of sustaining a firearm injury while on duty. One in five EMS clinicians in our sample said they have had a firearm drawn or used against them during a call, and three-quarters were at least somewhat worried about being injured by a firearm while on duty. Although health care practitioners experience disproportionately high rates of workplace violence compared to other professions, [44] EMS clinicians face unique risks as they see patients in a varied and uncontrolled environment. It is therefore essential that firearm injury prevention training address how EMS clinicians can keep themselves, their crew, and their patients safe when firearms are present. Several participants called for additional personal protective equipment (e.g., body armor) and training on how to disarm a patient in their write-in responses, and nearly half (45.7%) expressed interest in learning how to properly handle a firearm and render it safe. Training on safe handling and disarming of firearms could be offered as an optional training opportunity for EMS personnel. This training should cover local policies, procedures, and guidelines and could involve law enforcement as instructors.

Second, our findings suggest that EMS clinicians may have more personal experience with firearms than physicians. Approximately 70% of participants reported currently or previously owning a firearm, which is similar to estimates found in other studies examining firearm ownership among EMS clinicians [31, 33, 45]. Moreover, 73% of EMS clinicians in our study said they had taken a firearms safety course, and over 80% reported feeling comfortable handling a firearm. These findings align with a survey of EMS clinicians in West Texas, where 74% reported at least moderate experience with firearms (i.e., have used firearms many times, feel comfortable using them safely and effectively), and 78% said they felt safe handling, clearing, or engaging the safety system of a firearm encountered during patient care [31]. These numbers contrast sharply with rates of firearm ownership and exposure among physicians. In a survey of 218 medical residents and fellows in California, 14% reported owning a firearm and 22% had taken a firearms safety course [34]. Although rates of firearm ownership may be higher among physicians elsewhere in the US (California has a below-average rate of firearm ownership), estimates are still substantially lower than those among EMS clinicians [36, 46].

Due to their greater familiarity with firearms, EMS clinicians, as a group, may possess a higher level of cultural competence in this area than other health care practitioners, [47] positioning them as trusted messengers for firearm safety information. According to a 2016 nationally representative survey, US firearm owners perceive law enforcement and veterans as some of the most effective groups to teach firearm owners about safe firearm storage practices, and physicians as one of the least effective [48]. Based on their rates of firearm ownership and training in the safe handling and use of firearms, EMS clinicians may fall somewhere in between – well-positioned to connect authentically with firearm-owning patients and discuss firearm safety in a way that feels respectful, relatable, and grounded in shared life experience. Research also suggests that health care practitioners who own guns may be more likely than those who do not to counsel their patients about firearm safety, even if they are less likely to express support for the idea of counseling [46]. Support for gun ownership in participants’ qualitative responses suggests that the acceptability of clinician-led firearm safety counseling hinges on respecting the right to own firearms and framing conversations around health and safety rather than personal opinions or politics. However, because firearm owners are a heterogeneous group, [6, 49] having shared firearms experience is not enough to make EMS clinicians effective messengers of firearm injury prevention. To provide the best possible care to their patients, EMS clinicians should practice cultural humility – a national standard in EMS education – whether they own firearms or not [37].

Contrary to our expectations, over 60% of EMS clinicians reported receiving some type of medical education on firearm injury prevention, including 50% who said it was addressed in their primary education program (e.g., EMT or paramedic classes). This is somewhat surprising given that firearm injury prevention is not a core topic covered in EMS education programs [37]. However, we did not directly ask about the breadth or depth of material covered in participants’ previous coursework. Based on the existing literature and participants’ qualitative responses, it seems likely that such education primarily focuses on EMS response to active shootings and other firearm violence incidents (e.g., assessing scene safety, treating gunshot victims, collaborating with law enforcement). In another study of EMS clinicians from different parts of the US, only 13% reported receiving any specific training on assessing firearm ownership or access, and 7% reported receiving training on discussing secure firearm storage practices with patients [45]. In the current study, responses such as “We respond after something happens” and “This is what law enforcement is for” further highlight the perception (and arguably the misconception) that EMS providers have a solely reactive role in firearm-related incidents.

This narrower perspective misses the opportunities EMS clinicians may have to reduce the risk of firearm-related injury and death. Indeed, a notable share of EMS clinicians in our study reported having encountered different firearm risk situations on duty when law enforcement was not present, including calls in which firearms were accessible to children, to suicidal patients, or in domestic violence situations. In addition, more than half of participants said they had located a firearm on an altered or unresponsive patient, and 44% said they had handled a firearm while on duty and in the absence of law enforcement. These findings, and similar findings from prior research, highlight the need for expanded EMS training on how to identify patients at increased risk of firearm injury and strategies for reducing risk, including but not limited to safe storage counseling and ways to safely and legally remove firearms from the home during a crisis [31, 32]. Training should also address protocols and policies for dealing with firearms on scene, both when law enforcement are and are not present, as research suggests that what happens in practice frequently differs from what is expected [31].

The vast majority of participants (84.6%) expressed interest in additional education and training on firearm injury prevention, particularly on interventions. The BulletPoints Project offers an array of training materials and resources, including a free, online continuing education course, for clinicians interested in incorporating firearm injury prevention into their practice [20]. However, given the differences in their experience with firearms both personally and in the field, EMS clinicians may benefit from additional materials tailored to their unique needs and experiences.

### Limitations

Findings should be interpreted in light of some limitations, namely those related to generalizability and potential non-response, recall, and social desirability biases. Because this study utilized a nonprobability, convenience sample and nearly three-quarters of participants were living in California, the findings may not be representative of the experiences and perspectives of EMS clinicians at large. Furthermore, we were not able to calculate a response rate because we do not know how many individuals received the survey from the EMS departments and groups that we contacted. Of those who responded and were eligible, 66.5% completed the survey, which may further bias the results. More specifically, our sample may have consisted of individuals with greater interest or experience in firearm injury prevention than non-respondents or those who did not complete the survey. As with most survey studies, findings may also be subject to recall and social desirability biases. However, we tried to minimize these risks by making the survey anonymous and by using a short recall period (i.e., three months) for questions about the frequency of events and binary (yes/no) measures for questions about lifetime or career experiences.

## Conclusions

Although EMS clinicians typically respond after an emergency or crisis occurs, with appropriate education and training, their unique prehospital perspective and skills can also be leveraged to help prevent firearm injuries before they occur. To maximize acceptability and uptake, it is important for training curricula for EMS clinicians to: (1) respect the right to own firearms and frame conversations about firearms within the context of health and safety, not politics; (2) prioritize a risk-based approach to firearms screening that complements other risk assessment procedures; (3) highlight interventions that EMS clinicians can implement to reduce firearm injury risk, such as safe storage counseling; (4) address the increased risk of firearm injury among EMS clinicians while on duty; and (5) align with local policies and procedures, including those for when law enforcement are and are not present. Expanding EMS clinicians’ understanding, confidence, and competence in prevention efforts, including firearm injury prevention, will enhance the safety of first responders and our communities.

## Electronic supplementary material

Below is the link to the electronic supplementary material.

Supplementary Material 1: Appendix: Survey instrument

## Data Availability

The data that support the findings of this study are available from the corresponding author upon reasonable request.

## Abbreviations

CCW: Carry concealed weapon
EMS: Emergency medical services
ERPO: Extreme risk protection order
IPV: Intimate partner violence
LMSC: Lethal means safety counseling
PD: Police department
US: United States
