# Supplementary material for: Experiences with and perspectives on firearm injury prevention among emergency medical services clinicians
Source: BMC Emerg Med. 2025 Jun 2;25:88. doi: 10.1186/s12873-025-01241-9 (PMC12131716; doi:10.1186/s12873-025-01241-9)
Supplement: Supplementary file 1 — Supplementary Material 1: Appendix: Survey instrument [file 12873_2025_1241_MOESM1_ESM.docx]

**Appendix. Survey Instrument**

**SCREENER QUESTION**

Please think back on the last 3 months. In the last months, did you work 5 or more shifts as an EMS provider?

- Yes, I worked 5 or more shifts as an EMS provider in the last 3 months 🡪 CONTINUE TO CONSENT
- No, I did not work 5 or more shifts as an EMS provider in the last 3 months 🡪 EXIT

**SURVEY QUESTIONS**

1. Among the patients you serve, what type of firearm-related harm is most concerning? **MARK ONLY ONE**.
2. Homicide or interpersonal violence
3. Suicide or self-harm
4. Unintentional injury

1. **In the last 3 months**, approximately what percentage of your calls involved a patient or other person who was *at risk of* causing or sustaining a firearm-related injury?
2. None
3. A small amount (1-25%)
4. A moderate amount (26-50%)
5. Many of them (51-75%)
6. Most of them (76-100%)

1. **In the last 3 months**, during approximately what percentage of your calls did you ask your patients *who were at risk* about their access to firearms or the presence of firearms?
2. None
3. A small amount (1-25%)
4. A moderate amount (26-50%)
5. Many of them (51-75%)
6. Most of them (76-100%)

1. Which of the following are reasons you would **NOT** ask an at-risk patient about their access to firearms? **MARK ALL THAT APPLY**.
2. I don’t have time to ask.
3. I’m worried that the patient will be offended if I ask.
4. I don’t think firearm access is relevant to their risk of injury or death.
5. I’m not sure that it’s legal for me to ask.
6. I don’t think asking about firearm access is within my professional responsibilities.
7. I don't know how to ask about firearm access.
8. I wouldn’t know what to do if the patient had a gun.
9. There’s nothing I could do for an at-risk patient who has a gun.

1. **In the last 3 months**, during approximately what percentage of your calls did you provide education or counseling about firearm injury prevention to your patients *who were at risk*?
2. None
3. A small amount (1-25%)
4. A moderate amount (26-50%)
5. Many of them (51-75%)
6. Most of them (76-100%)

1. How comfortable are you counseling your patients about firearm injury prevention?
2. Very comfortable
3. Comfortable
4. Uncomfortable
5. Very uncomfortable

1. Please indicate if you have **ever** been on duty in the following situations when law enforcement was **not** present.
2. I have had a firearm drawn on me or used against me.

- Yes
- No

1. I have been physically assaulted by a patient.

- Yes
- No

1. I have handled a firearm during a call.

- Yes
- No

1. I have been on a call in which an at-risk patient had access to a firearm.

- Yes
- No

1. I have located a firearm in the possession of an altered or unresponsive patient.

- Yes
- No

1. I have cared for a patient presenting with active suicidal ideation or following a suicide attempt who had firearm access.

- Yes
- No

1. I have been present during a domestic violence situation in which a firearm was present.

- Yes
- No

1. I have been on a call where firearms were accessible to children.

- Yes
- No

1. In general, when you’re on duty, how worried are you that a patient or someone else might injure you with a firearm (on purpose or on accident)?
2. Not worried at all
3. Somewhat worried
4. Worried
5. Very worried

1. Which of the following best describes your experience with firearms? MARK **ALL THAT APPLY**.
2. I own or have owned a firearm
3. Someone I live with owns a firearm but I don’t
4. I grew up around firearms
5. I’ve used a firearm before
6. I’ve never used a firearm
7. I used a firearm as part of my military service
8. Prefer not to answer

1. Have you ever taken a course about the safe handling and use of firearms?
2. Yes
3. No

1. How comfortable are you handling a firearm?
2. Very comfortable
3. Comfortable
4. Uncomfortable
5. Very uncomfortable

1. How interested are you in an educational program designed to increase your knowledge and skills in counseling patients about firearm injury prevention?
2. Very interested
3. Interested
4. Slightly interested
5. Not interested at all

1. Which of the following would you be interested in learning more about? **MARK ALL THAT APPLY**.
2. How to identify patients at risk of firearm-related injury
3. How to talk with patients about their access to firearms
4. How to counsel patients on safe storage of firearms
5. How to properly handle a firearm and render it safe
6. Why patients would be prohibited from owning or purchasing firearms
7. What to do if at-risk patients are in possession of firearms
8. Evidence-based interventions to prevent firearm injury and death in patients

1. What other firearm injury prevention topics would you be interested in learning about?

[WRITE IN]

1. How old are you?
2. 24 or younger
3. 25 – 34
4. 35 – 44
5. 45 – 54
6. 55 or older

1. What is your gender?
2. Man
3. Woman
4. Other (please specify): __________
5. Prefer not to answer

1. In what state did you graduate high school? (This gives us an idea of where you grew up.)

  [DROP DOWN LIST]

1. What state do you live in?

  [DROP DOWN LIST]

1. What is the **primary** service for which you work or volunteer as an EMS provider? **MARK ONLY ONE.**
2. Hospital-based EMS service
3. Fire Department-based EMS service
4. Private EMS service
5. Volunteer-based EMS service
6. Other (please specify): ___________

1. What is your **highest level** of medical certification? **MARK ONLY ONE.**
2. First Responder
3. Emergency Medical Technician (EMT)
4. Advanced Emergency Medical Technician (AEMT)
5. Paramedic
6. Registered Nurse (RN)
7. Physician Assistant (PA)
8. Physician
9. None of the above

1. When has firearm-related injury prevention been addressed in your medical education? **MARK ALL THAT APPLY**.
2. EMT, AEMT, or Paramedic EMS courses
3. RN, PA, or Physician training
4. Continuing education courses
5. Other professional education (please specify): ___________
6. Never

1. Is there anything else you’d like to share with us?

[WRITE IN]
